# Immunity passports, fundamental rights and public health hazards: a reply to Brown *et al*

**DOI:** 10.1136/medethics-2020-106814

**Published:** 2020-09-09

**Authors:** Iñigo de Miguel Beriain, Jon Rueda

**Affiliations:** 1 Derecho Publico, UPV/EHU, Bilbao, Spain; 2 Ikerbasque, Bilbao, Spain; 3 Department of Philosophy 1, University of Granada, Granada, Spain

**Keywords:** ethics, law, public policy, rights

## Abstract

In their recent article, Brown *et al* analyse several ethical aspects around immunity passports and put forward some recommendations for implementing them. Although they offer a comprehensive perspective, they overlook two essential aspects. First, while the authors consider the possibility that immunological passports may appear to discriminate against those who do not possess them, the opposite viewpoint of immune people is underdeveloped. We argue that if a person has been tested positive for and recovered from COVID-19, becoming immune to it, she cannot be considered a hazard to public health and, therefore, the curtailment of her fundamental rights (eg, the right to freedom of movement) is not legitimate. Second, they omit that vaccine distribution will create similar problems related to immunity-based licenses. Vaccine certificates will de facto generate a sort of immunity passport. In the next phases of the pandemic, different immunity statuses will be at stake, because the need to identify who can spread COVID-19 is unavoidable. If a person does not pose a threat to public health because she cannot spread the infection, then her right to freedom of movement should be respected, regardless of how she acquired that immunity.

The literature on the ethical questions raised by so-called immunity passports has grown steadily in the last months.^1–6^ The recent article by Brown *et al* offers an interesting standpoint on the topic by analysing some of the practical considerations and prominent ethical aspects involved.^7^ However, in our view, it neglects two essential issues of the debate. First, the perspective of defending the fundamental rights of those who are immune to COVID-19 needs to be explored much more thoroughly. Second, immunity passports will de facto exist as soon as vaccines become available. These critical omissions have been similarly ignored by most commentators. The discussion should, however, focus on what kind of immunity statuses are legitimate in order to grant immunity-based licences and, equally importantly, what rights and liberties these statuses will entitle.

With regard to the first issue, it is necessary to refer to the Convention for the Protection of Human Rights and Fundamental Freedoms, signed in Rome on 4 November 1950. This is a fundamental legal tool that defends individual rights and freedoms in all signatory countries, including most European countries. According to article 5, ‘Everyone has the right to liberty and security of person. No one shall be deprived of his liberty save in the following cases and in accordance with a procedure prescribed by law: (…) (*e*) the lawful detention of persons for the prevention of the spreading of infectious diseases, of persons of unsound mind, alcoholics or drug addicts or vagrants’.^8^

The Convention establishes a right to freedom of movement that cannot be restricted but ‘for the prevention of the spreading of infectious diseases’. It is necessary to emphasise that the Convention does not even allow in principle to limit the freedom of movement to avoid a risk of collapse for the health system—one of the reasons given to justify the lockdowns, as Brown *et al* pointed out. The question according to the Convention is simply whether a citizen poses a threat to public health. Moreover, the European Court of Human Rights determined in the case of Enhorn v. Sweden (application no. 56529/00 of 25 January 2005) that

the essential criteria when assessing the ‘lawfulness’ of the detention of a person ‘for the prevention of the spreading of infectious diseases’ are whether the spreading of the infectious disease is dangerous to public health or safety, and whether detention of the person infected is the last resort in order to prevent the spreading of the disease, because less severe measures have been considered and found to be insufficient to safeguard the public interest. When these criteria are no longer fulfilled, the basis for the deprivation of liberty ceases to exist.^9^

This articulation of ‘lawfulness’ should be the key criterion in the entire process of awarding immunity passports: whether the deprivation of liberty is the only way to safeguard public interest.

A relevant question is who should determine public interest, which becomes the heart of the matter. Normally, the entity who wants to deprive a right (eg, the health authority) should provide evidence; in this situation, evidence that only curtailing the right to freedom of movement can prevent the spread of the disease. What is extraordinary about COVID-19 is that it has allowed governments to establish a presumption of hazard for entire populations. This hazard, governments claim, can primarily be neutralised through lockdowns.^10^

Can this presumption be maintained in the future? This is where the emergence of vaccines as game-changers comes into play: as soon as new vaccines are approved and begin to be administered, there will be a profound discussion about whether vaccines will guarantee greater rights and freedoms to those who are vaccinated than to others. This may not be the case. If not, vaccine certificates would not serve as immunity passports. One could maintain the idea that those vaccinated would be people working in vital sectors (eg, healthcare, food supply chain workers, transporters, police and social workers) and people especially vulnerable to COVID-19. A plausible justification would be to stop the chains of contagion in the former and not expose the latter to serious health risks, without conferring them a privileged immunity status. Yet, this position would be hard to maintain both for scientific reasons—it is difficult to sustain the hypothesis that vaccines do not confer immunity—and for practical reasons—it will not be easy to keep vaccinated groups confined due to public health reasons such as the peril of spreading COVID-19.

Our hypothesis, by contrast, is that society will be quick to consider that those who receive the vaccines are immune to COVID-19. Therefore, vaccinated people will reject being deprived of their basic rights and freedoms. Ultimately, this effect of dividing society into two large groups, the seropositive and the seronegative, will be unavoidable, even if we do not issue immunity passports to those who have recovered from COVID-19 without receiving the vaccine.

Another question to consider is why, in the case of vaccine certificates, this scenario does not create for us the same perplexity as other types of acquisition of immunity against COVID-19—either immunity acquired by contagion or by individuals who have not been exposed to SARS-CoV-2 but who possess efficient T-lymphocytes leading to cross-immunity. In principle, one might think that there are notable differences in that the vaccine creates a different form of immunity. This, however, is not sound. In the short term, we will not know exactly what the effects of the vaccine are and whether they are the same for every recipient. In this regard, we will maintain a similar degree of uncertainty between one method of acquiring immunity (ie, vaccines) and another (ie, antibodies following recovery). Moreover, if we accept that the vaccine produces some form of immunity, it will be because we have been able to establish some test that will allow us to certify that immunity. If such a test exists, it would be logical to respect the right to freedom of movement for all persons who satisfy that test, regardless of how they have acquired that immunity.

Obviously, administering this test to everyone would be expensive and unnecessary in the case of those who have been vaccinated, because we should assume the overall success of the immunisation process. Evidence of immunity for those infected or for others who might try to claim immunity would instead be necessary, which would still involve a cost. A health system may not be able to assume the total amount of massive antibody tests. This reasoning, however, is somewhat misleading. To begin with, it is problematic to take tests into account if the affected person covers the cost of the test; Brown *et al* note that ‘[a]ccess to testing should not, therefore, rely on personal wealth’. Still, this concern does not preclude the crucial question: if a person is able to show clinical evidence that she meets the standard immunity requirements, on what grounds could she be denied further enjoyment of her right to freedom of movement?

There are only two good reasons to counter this freedom. The first is that it would create unfair discrimination to not allow all those who have potentially been infected or naturally immune to be tested. This is a problem of distributive justice, which must not be solved by depriving some people of the possibility of regaining their fundamental rights—that is, avoiding a levelling down move.^2^ The second is that, unlike vaccines, the acquisition of immunity by contagion does not require financial capabilities. This would encourage the pernicious effect of people voluntarily exposing themselves to become infected in order to regain their fundamental rights. But again, these deleterious possibilities are not a reason to maintain unjust forced confinement on those who do not pose a threat to public health; rather, it is a reason to mitigate free-riding and reduce the incentives for contagion.

In light of these reflections, we must conclude that, in fact, it will be necessary to differentiate (even if it seems discriminatory) between people who are immune and not immune to COVID-19 for public health reasons. Moreover, there are not legal reasons to discriminate those who cannot spread the disease. The basic issue will always be the same: if she is immune, there is no legal basis to curtail her fundamental rights, especially the right to freedom of movement. If not, there would be reasons for that curtailment. So there will indeed be two different types of citizens. Hopefully, this division will be short-lived and a vaccine will become available to large masses of people within an acceptable timeframe (say, for instance, 1 year). However, a further pressing problem may arise: those who do not want to be vaccinated will probably form a separate and perhaps discriminated-against population group. Another heated discussion will emerge about how to deal with this challenging issue. Unfortunately, we do not have space to address this debate here.
